# Editorial: Variability and reproducibility of brain imaging

**DOI:** 10.3389/fpsyg.2024.1386948

**Published:** 2024-03-13

**Authors:** Meng-Yun Wang, Helge J. Zöllner, Meryem A. Yücel, Karsten Specht

**Affiliations:** ^1^Department of Biological and Medical Psychology, University of Bergen, Bergen, Norway; ^2^Mohn Medical Imaging and Visualization Centre (MMIV), Department of Radiology, Haukeland University Hospital, Bergen, Norway; ^3^The Russell H. Morgan Department of Radiology and Radiological Science, Johns Hopkins University School of Medicine, Baltimore, MD, United States; ^4^F.M. Kirby Research Center for Functional Brain Imaging, Kennedy Krieger Institute, Baltimore, MD, United States; ^5^Neurophotonics Center, Department of Biomedical Engineering, Boston University, Boston, MA, United States; ^6^Department of Education, UiT The Arctic University of Norway, Tromsø, Norway

**Keywords:** neuroimaging, reliability, fMRI, MRS, variability, replication crisis

The human brain processes a constant stream of sensory information, necessitating advanced filtering and cognitive processing for tasks like recognizing dynamic facial expressions (Wang and Yuan, 2021). Advances in noninvasive neuroimaging methods, such as electroencephalogram (EEG) (Wang et al., 2020a), magnetic resonance imaging (MRI) (Lu et al., 2021), and near-infrared spectroscopy (NIRS) (Wang et al., 2020b) have deepened our understanding of how the human brain works over the last decades (Finn et al., 2023). However, the reliability and reproducibility of the results have been recently questioned and exposed in the spotlight (Cognitive neuroscience at the crossroads, 2022), highlighting the need to address and clarify the integrity of the research field (Revisiting doubt in neuroimaging research, 2022).

Hence, the main goal of this Research Topic is to address and explore which factors influence the reliability and reproducibility of brain imaging results and provide practical perspectives and insights to enhance them. We have collected four articles for the Research Topic, whose contributions were discussed as follows.

The first article explored the contributing factors to the brain age index. Brain age is the age gap between chronological age and predicated age from brain imaging data (Cole and Franke, 2017). There is evidence showing that brain age is associated with common brain disorders (Kaufmann et al., 2019), and it has been considered as a potential biomarker for various psychiatric disorders (Cole and Franke, 2017). However, the contribution to brain age from non-brain imaging factors besides neuroimaging data has not been explored. Korbmacher et al. have explored this issue and proposed a Bio-psycho-social model for predicting brain age. The study utilized the UK Biobank data (Miller et al., 2016) and compared different model configurations. They discovered that the Bio-psycho-social model can partially explain the brain age variance which is comparable to the contribution of the diffusion MRI approach. Additionally, they found large variability in gender and ethnicity differences in brain age. They then suggest that in future studies, the effects of ethnicity, cognitive factors, gender, as well as health and lifestyle factors should be controlled to observe the bio-psycho-social factor impacts on brain age.

The second article touched upon the reliability of the resting-state (rs-)fMRI. Put simply, rs-fMRI captures systematic, non-random variations in brain activation in the absence of a specific task. These activations are indirectly reflected in regional fluctuations in the level of oxygenation of the blood (known as BOLD) (Ogawa et al., 1992), which has been widely used to explore the functional brain activation (Lu et al., 2020) and brain networks (Damoiseaux et al., 2006; Bullmore and Sporns, 2009; van den Heuvel and Pol, 2010). There is evidence suggesting that the time-of-day could be associated with reduced global signal fluctuation and functional connectivity (Orban et al., 2020) alongside the time-of-year effect (Wang et al., 2023; Zhang et al., 2023). However, it's still unclear how the time-of-day effect, along with other factors such as age and gender, could sway the results of longitudinal neuroimaging data. Vaisvilaite et al. addressed this issue by leveraging the BETULA dataset, a longitudinal study on aging (Nilsson et al., 1997), and employing the cross-spectral dynamic causal modeling (DCM) (Friston et al., 2003), which puts the temporal fluctuations of resting-state BOLD signal into the focus. Specifically, they explored eight DCM models and found that the time-of-day effect together with gender significantly influences the parameters defining the BOLD signal. Therefore, they suggest that when collecting longitudinal neuroimaging datasets, time of day should be considered a covariate in addition to age and gender.

The third article discussed the reliability of the magnetic resonance spectroscopy (MRS) measurement in multi-site/vendor studies. MRS allows for non-invasive investigation of neurometabolic profiles in health and disease, yielding metabolite estimates from a localized volume (Oz et al., 2014; Wilson et al., 2019). Widely used in clinical settings (Oz et al., 2014), MRS has recently been applied to identify IDH-mutated gliomas (Branzoli and Marjanska, 2020) and explore the association between behavior and MRS-derived neurotransmitters (Li et al., 2022). It is suggested that the MRS-derived metabolite estimates are reliable within the participants (Wang et al., 2024). In large-scale clinical studies, a multi-site/vendor design is often favored to achieve the necessary statistical power. However, the impact of different sites and scanners on metabolite estimates is frequently overlooked (Považan et al., 2020). La et al. investigated how to account for these differences in a pediatric concussion dataset comprising 545 short-TE MRS measurements, collected across six MRI scanners with two MRI vendors at five scanning sites. They used four general linear models and three linear mixed-effects models to investigate site- and vendor effects on the quantitative estimates of five major neuro-metabolites. They found that different analysis strategies of controlling site, vendor, and scanner in MRS data generated different results, from which they advocate that the ComBat harmonization for clinical MRS data (Bell et al., 2022) should be utilized to remove the site and vendor effects.

The fourth article is even more clinically related, exploring the repeatability of 2D and 4D flow MRI measurement of intracranial blood flow and pulsatility. Arterial stiffening impacts blood vessel compliance and regulation and is considered a contributing factor to various neurological conditions (Poels et al., 2012). To noninvasively examine arterial stiffening specifically the blood flow velocity, 2D phase-contrast MRI is dominantly used (McCauley et al., 1995). However, it can only measure a few vessels per scan. On the contrary, the 4D flow MRI can evaluate multiple vessels simultaneously, making it an attractive alternative (Terada et al., 2022). Yet, the repeatability, reliability, and consistency of 4D flow compared to 2D across intracranial vessels remain uncertain. In the study, Morgan et al. aimed to answer this scientific inquiry. They used various statistical methods including intra-rater reliability, inter-method conformity, and test-retest repeatability to assess the repeatability of the pulsatility index and mean flow among patients with small vessel disease and healthy controls. They found that the 4D flow MRI possesses repeatable and reliable PI measurements and the mean blood flow measurement. Therefore, they have suggested the 4D flow MRI can be confidently used to assess the pulsatility across the major cerebral vessels.

We appreciate the contributions of the authors of these four articles, which hopefully could stimulate and elicit further discussions about the topic: the variability and reliability of brain imaging. Indeed, we have witnessed collective initiatives to enhance the robustness of the neuroimaging results (Korbmacher et al., 2023), such as best practices in the data analysis (Nichols et al., 2017; Boudewyn et al., 2023), guidelines for reporting or publishing (Poldrack et al., 2008; Keil et al., 2014; Lin et al., 2021; Yücel et al., 2021), and expert consensus (Choi and Kreis, 2021). We firmly believe by acknowledging the problems we have in neuroimaging studies and adopting the best practices, the neuroimaging results will be more robust and reliable.

## Author contributions

M-YW: Writing—original draft, Writing—review & editing, Conceptualization. HZ: Writing—review & editing, Conceptualization. MY: Writing—review & editing, Conceptualization. KS: Writing—review & editing, Conceptualization.

## References

[B1] Bell T. K. Godfrey K. J. Ware A. L. Yeates K. O. Harris A. D. (2022). Harmonization of multi-site MRS data with ComBat. Neuroimage 257:119330. 10.1016/j.neuroimage.2022.119330 35618196

[B2] Boudewyn M. A. Erickson M. A. Winsler K. Ragland J. D. Yonelinas A. Frank M.. (2023). Managing EEG studies: how to prepare and what to do once data collection has begun. Psychophysiology 60:e14365. 10.1111/psyp.14365 37314113 PMC11276027

[B3] Branzoli F. Marjanska M. (2020). Magnetic resonance spectroscopy of isocitrate dehydrogenase mutated gliomas: current knowledge on the neurochemical profile. Curr. Opin. Neurol. 33, 413–421. 10.1097/WCO.0000000000000833 32657882 PMC7526653

[B4] Bullmore E. T. Sporns O. (2009). Complex brain networks: graph theoretical analysis of structural and functional systems. Nat. Rev. Neurosci. 10, 186–198. 10.1038/nrn2575 19190637

[B5] Choi I. Y. Kreis R. (2021). Advanced methodology for *in vivo* magnetic resonance spectroscopy. NMR Biomed. 34:e4504. 10.1002/nbm.4504 33709530

[B6] Cognitive neuroscience at the crossroads. (2022). Nature 608:647. 10.1038/d41586-022-02283-w 36002501

[B7] Cole J. H. Franke K. (2017). Predicting age using neuroimaging: innovative brain ageing biomarkers. Trends Neurosci. 40, 681–690. 10.1016/j.tins.2017.10.001 29074032

[B8] Damoiseaux J. S. Rombouts S. A. Barkhof F. Scheltens P. Stam C. J. Smith S. M.. (2006). Consistent resting-state networks across healthy subjects. Proc. Natl Acad. Sci. USA. 103, 13848–13853. 10.1073/pnas.0601417103 16945915 PMC1564249

[B9] Finn E. S. Poldrack R. A. Shine J. M. (2023). Functional neuroimaging as a catalyst for integrated neuroscience. Nature 623, 263–273. 10.1038/s41586-023-06670-9 37938706

[B10] Friston K. J. Harrison L. Penny W. (2003). Dynamic causal modelling. Neuroimage 19, 1273–1302. 10.1016/S1053-8119(03)00202-7 12948688

[B11] Kaufmann T. van der Meer D. Doan N. T. Schwarz E. Lund M. J. Agartz I.. (2019). Common brain disorders are associated with heritable patterns of apparent aging of the brain. Nat. Neurosci. 22, 1617–1623. 10.1038/s41593-019-0471-7 31551603 PMC6823048

[B12] Keil A. Debener S. Gratton G. Junghöfer M. Kappenman E. S. Luck S. J.. (2014). Committee report: publication guidelines and recommendations for studies using electroencephalography and magnetoencephalography. Psychophysiology 51, 1–21. 10.1111/psyp.12147 24147581

[B13] Korbmacher M. Azevedo F. Pennington C. R. Hartmann H. Pownall M. Schmidt K.. (2023). The replication crisis has led to positive structural, procedural, and community changes. Commun. Psychol. 1:3. 10.1038/s44271-023-00003-2 PMC11290608 39242883

[B14] Li H. Heise K.-F. Chalavi S. Puts N. A. J. Edden R. A. E. Swinnen S. P.. (2022). The role of MRS-assessed GABA in human behavioral performance. Prog. Neurobiol. 212:102247. 10.1016/j.pneurobio.2022.102247 35149113

[B15] Lin A. Andronesi O. Bogner W. Choi I.-Y. Coello E. Cudalbu C.. (2021). Minimum reporting standards for *in vivo* magnetic resonance spectroscopy (MRSinMRS): experts' consensus recommendations. NMR Biomed. 34:e4484. 10.1002/nbm.4484 33559967 PMC8647919

[B16] Lu F. Liu P. Chen H. Wang M. Xu S. Yuan Z.. (2020). More than just statics: abnormal dynamic amplitude of low-frequency fluctuation in adolescent patients with pure conduct disorder. J. Psychiatr. Res. 131, 60–68. 10.1016/j.jpsychires.2020.08.027 32937251

[B17] Lu F. Wang M. Xu S. Chen H. Yuan Z. Luo L.. (2021). Decreased interhemispheric resting-state functional connectivity in male adolescents with conduct disorder. Brain Imaging Behav. 15, 1201–1210. 10.1007/s11682-020-00320-8 32623563

[B18] McCauley T. R. Peña C. S. Holland C. K. Price T. B. Gore J. C. (1995). Validation of volume flow measurements with cine phase-contrast MR imaging for peripheral arterial waveforms. J. Magn. Reson. Imaging 5, 663–668. 10.1002/jmri.1880050608 8748483

[B19] Miller K. L. Alfaro-Almagro F. Bangerter N. K. Thomas D. L. Yacoub E. Xu J.. (2016). Multimodal population brain imaging in the UK Biobank prospective epidemiological study. Nat. Neurosci. 19, 1523–1536. 10.1038/nn.4393 27643430 PMC5086094

[B20] Nichols T. E. Das S. Eickhoff S. B. Evans A. C. Glatard T. Hanke M.. (2017). Best practices in data analysis and sharing in neuroimaging using MRI. Nat. Neurosci. 20, 299–303. 10.1038/nn.4500 28230846 PMC5685169

[B21] Nilsson L. G. Adolfsson R. Bäckman L. de Frias C. Molander B. Nyberg L.. (1997). The Betula prospective cohort study: memory, health and aging. Aging Neuropsychol. Cogn. 4, 1–32. 10.1080/13825589708256633

[B22] Ogawa S. Tank D. W. Menon R. Ellermann J. M. Kim S. G. Merkle H.. (1992). Intrinsic signal changes accompanying sensory stimulation - functional brain mapping with magnetic-resonance-imaging. Proc. Natl. Acad. Sci. USA. 89, 5951–5955. 10.1073/pnas.89.13.5951 1631079 PMC402116

[B23] Orban C. Kong R. Li J. Chee M. W. L. Yeo B. T. T. (2020). Time of day is associated with paradoxical reductions in global signal fluctuation and functional connectivity. Plos Biol. 18:e3000602. 10.1371/journal.pbio.3000602 32069275 PMC7028250

[B24] Oz G. Alger J. R. Barker P. B. Bartha R. Bizzi A. Boesch C.. (2014). Clinical proton MR spectroscopy in central nervous system disorders. Radiology 270, 658–679. 10.1148/radiol.13130531 24568703 PMC4263653

[B25] Poels M. M. Zaccai K. Verwoert G. C. Vernooij M. W. Hofman A. van der Lugt A.. (2012). Arterial stiffness and cerebral small vessel disease the rotterdam scan study. Stroke 43, 2637–2642. 10.1161/STROKEAHA.112.676528 22879099

[B26] Poldrack R. A. Fletcher P. C. Henson R. N. Worsley K. J. Brett M. Nichols T. E.. (2008). Guidelines for reporting an fMRI study. Neuroimage 40, 409–414. 10.1016/j.neuroimage.2007.11.048 18191585 PMC2287206

[B27] Považan M. Mikkelsen M. Berrington A. Bhattacharyya P. K. Brix M. K. Buur P. F.. (2020). Comparison of multivendor single-voxel MR spectroscopy data acquired in healthy brain at 26 sites. Radiology 295, 171–180. 10.1148/radiol.2020191037 32043950 PMC7104702

[B28] Revisiting doubt in neuroimaging research (2022). Nat. Neurosci. 25, 833–834. 10.1038/s41593-022-01125-2 35790858

[B29] Terada M. Takehara Y. Isoda H. Wakayama T. Nozaki A. (2022). Technical background for 4D flow MR imaging. Magn. Reson. Med. Sci. 21, 267–277. 10.2463/mrms.rev.2021-0104 35153275 PMC9680548

[B30] van den Heuvel M. P. Pol H. E. H. (2010). Exploring the brain network: a review on resting-state fMRI functional connectivity. Eur. Neuropsychopharmacol. 20, 519–534. 10.1016/j.euroneuro.2010.03.008 20471808

[B31] Wang M.-Y. Yuan A. Zhang J. Xiang Y. Yuan Z. (2020b). Functional near-infrared spectroscopy can detect low-frequency hemodynamic oscillations in the prefrontal cortex during steady-state visual evoked potential-inducing periodic facial expression stimuli presentation. Vis. Comput. Ind. Biomed. Art 3:28. 10.1186/s42492-020-00065-7 33258067 PMC7704826

[B32] Wang M.-Y. Zhang Z.-M. Miao X. Xiaohong X. Yuan Z. (2020a). Electrophysiological evidence of attentional avoidance in sub-clinical individuals with obsessive-compulsive symptoms. IEEE Access 8, 91020–91027. 10.1109/ACCESS.2020.2994452

[B33] Wang M. Y. Korbmacher M. Eikeland R. Craven A. R. Specht K. (2024). The intra-individual reliability of H-MRS measurement in the anterior cingulate cortex across 1 year. Hum. Brain Mapp. 45:e26531. 10.1002/hbm.26531 37986643 PMC10789202

[B34] Wang M. Y. Korbmacher M. Eikeland R. Specht K. (2023). “The long night effect on the brain functional organization, in *The Organization for Human Brain Mapping 2023 Annual Conference* (Montreal).

[B35] Wang M. Y. Yuan Z. (2021). EEG decoding of dynamic facial expressions of emotion: evidence from SSVEP and causal cortical network dynamics. Neuroscience 459, 50–58. 10.1016/j.neuroscience.2021.01.040 33556458

[B36] Wilson M. Andronesi O. Barker P. B. Bartha R. Bizzi A. Bolan P. J.. (2019). Methodological consensus on clinical proton MRS of the brain: review and recommendations. Magn. Reson. Med. 82, 527–550. 10.1002/mrm.27742 30919510 PMC7179569

[B37] Yücel M. A. Lühmann A. V. Scholkmann F. Gervain J. Dan I. Ayaz H.. (2021). Best practices for fNIRS publications. Neurophotonics 8:012101. 10.1117/1.NPh.8.1.012101 33442557 PMC7793571

[B38] Zhang R. Shokri-Kojori E. Volkow N. D. (2023). Seasonal effect-an overlooked factor in neuroimaging research. Transl. Psychiatry 13:238. 10.1038/s41398-023-02530-2 37400428 PMC10318079

